# Sex change in the subdioecious shrub *Eurya japonica* (Pentaphylacaceae)

**DOI:** 10.1002/ece3.2745

**Published:** 2017-03-10

**Authors:** Hui Wang, Michinari Matsushita, Nobuhiro Tomaru, Michiko Nakagawa

**Affiliations:** ^1^ School of Life ScienceShandong UniversityJinanChina; ^2^ Laboratory of Forest Ecology and PhysiologyGraduate School of Bioagricultural SciencesNagoya UniversityNagoyaJapan; ^3^ Forest Tree Breeding CenterForestry and Forest Products Research InstituteHitachiJapan

**Keywords:** internal physiological condition, sex expression, sex ratio, steady state, subdioecy

## Abstract

Sex change affects the sex ratios of plant populations and may play an essential role in the evolutionary shift of sexual systems. Sex change can be a strategy for increasing fitness over the lifetime of a plant, and plant size, environmental factors, and growth rate may affect sex change. We described frequent, repeated sex changes following various patterns in a subdioecious *Eurya japonica* population over five successive years. Of the individuals, 27.5% changed their sex at least once, and these changes were unidirectional or bidirectional. The sex ratio (females/males/all hermaphrodite types) did not fluctuate over the 5 years. In our study plots, although the current sex ratio among the sexes appears to be stable, the change in sex ratio may be slowly progressing toward increasing females and decreasing males. Sex was more likely to change with higher growth rates and more exposure to light throughout the year. Among individuals that changed sex, those that were less exposed to light in the leafy season and had less diameter growth tended to shift from hermaphrodite to a single sex. Therefore, sex change in *E*. *japonica* seemed to be explained by a response to the internal physiological condition of an individual mediated by intrinsic and abiotic environmental factors.

## 1 Introduction

Sex change in plants is thought to be a strategy for increasing fitness over the lifetime of a plant (Policansky, 1982). In most animal species in which it occurs, sex change occurs only once in an individual's lifetime (Munday, Buston, & Warner, 2006; Munday, Kuwamura, & Kroon, 2010), but many plants undergo repeated, multidirectional sex changes (Mirski, 2014; Nanami, Kawaguchi, & Yamakura, 2004; Schlessman, 1988; Ushimaru & Matsui, 2001; Yamashita & Abe, 2002). Such frequent sex changes affect the sex ratio of a population. Indeed, year‐by‐year fluctuations in sex ratios caused by sex change have been observed within a population (Nanami et al., 2004; Yamashita & Abe, 2002). Therefore, sex change may play an essential role in the evolutionary shift of a sexual system (Delph & Wolf, 2005; Spigler & Ashman, 2011).

Plant size (Yamashita & Abe, 2002), environmental factors (Freeman, Harper, & Charnov, 1980; Ghiselin, 1969), and growth rate (Nanami et al., 2004) all affect sex change. In some species, small plants reproduce as males, while larger plants reproduce as females (Bierzychudek, 1982; Kinoshita, 1987; Schlessman, 1991; Yamashita & Abe, 2002). Sex expression is sometimes correlated with environmental factors, such as light intensity and habitat condition. More females than males of an epiphytic orchid occur under open canopies (Zimmerman, 1991), and males have been observed to change to hermaphrodites under the best growing conditions on a moisture gradient (Sakai & Weller, 1991). Moreover, Nanami et al. (2004) found that sex change toward female occurred in unhealthy (i.e., slow growing) *Acer* trees after a decrease in precipitation. This observation suggests that sex change is regulated by the internal physiological condition of the plant itself (e.g., the availability of resources or health condition), which is affected by environmental circumstances (Matsui, 1995).

*Eurya japonica* Thunb. (Pentaphylacaceae) is an evergreen broadleaf subdioecious shrub. Tsuji and Sota (2013) reported that a single *E*. *japonica* individual changed from male to hermaphrodite bearing flowers of different sexes, while another shifted in the reverse direction; however, no study has examined the frequency and pattern of sex change or factors that affect sex change in *E*. *japonica*. In this study, we monitored sex expression in a subdioecious *E*. *japonica* population over five successive years, during which the growth rate and light condition of each individual were measured to (i) quantify the frequency and pattern of sex change, (ii) clarify the fluctuation in the sex ratio, and (iii) investigate the factors influencing the occurrence and pattern of sex change in *E*. *japonica*.

## 2 Material and Methods

### 2.1 Field survey

Three plots (Plots 1–3, each 20 × 20 m) were established in early February 2010 in a secondary forest on the Higashiyama Campus of Nagoya University, Japan (35°10′N, 136°58′E, 55–80 m a.s.l.; for details of the study site, see Wang, Matsushita, Tomaru, & Nakagawa, 2015). Plot 1 was destroyed for construction of a building in May 2012, and we added one plot (Plot 4, 20 × 20 m) at the Higashiyama Zoo and Botanical Gardens, Nagoya, Japan (35°09′N, 136°58′E, 79–86 m a.s.l.) in early February 2012. Plots 1 and 4 were ca. 1.1 km apart and were probably once covered by a single contiguous forest. The stand on the Higashiyama Campus was dominated by *Quercus variabilis* and *Q*. *serrata* (Fagaceae) in the canopy, and by *Q*. *glauca* (Fagaceae) and *E. japonica* in the lower vegetation (Hirano,^1993^; Makoto, 1991). The secondary forest in the Higashiyama Zoo and Botanical Gardens was dominated by *Q. variabilis* and *Ilex pedunculosa* (Aquifoliaceae) in the canopy, and by *Cleyera japonica* (Pentaphylacaceae), *Vaccinium bracteatum* (Ericaceae), and *E. japonica* in the lower vegetation. All *E*. *japonica* individuals >30 cm tall were tagged when each plot was established (a total of 74, 81, 102, and 52 individuals were monitored in the respective plots, respectively). We noted the flowering state (flowering vs. non‐flowering) of each individual in each spring and determined the sexes of all flowering individuals within the four plots by direct observation of the flowers from 10 shoots (about 20–25 cm in length, 17 ± 8 flowers per shoot) which were randomly selected from different locations on each individual. Individual plants were classified into six sexual categories: males bearing only staminate flowers (M), females with only pistillate flowers (F), hermaphrodites with only perfect flowers (H), hermaphrodites bearing a mixture of perfect and pistillate flowers (HF; 12 ± 8 pistillate flowers vs. 10 ± 6 perfect flowers; mean ± *SD* per shoot; *p* < .01), perfect and staminate flowers (HM; 9 ± 6 staminate flowers vs. 2 ± 2 perfect flowers; *p* < .001), and a mixture of all three flower types (HFM; 3 ± 2 pistillate flowers vs. 7 ± 6 staminate flowers vs. 6 ± 4 perfect flowers; *p* < .01). In February–March, we recorded the sex expression of each individual in plots 2 and 3 during 2010–2014, in Plot 1 during 2010–2012, and in Plot 4 during 2012–2014. The annual temperature and precipitation during our 5‐year study were similar to the average value for 1981–2010 provided by the Nagoya meteorological station (Fig. S1).

To test the effects of individual size, growth rate, and the light environment on sex change, we measured diameter at breast height (DBH) each March from 2010 to 2014. Each February (when deciduous plants are leafless) during 2011–2014 and in late June (leafy season when the upper layer is covered with foliage) during 2010–2013, the photosynthetic photon flux density (PPFD) was measured three times above the crown of each flowering individual using a quantum sensor (LI‐190SA, Li‐Cor Biosciences). Simultaneously, PPFD was measured in an open site to calculate the mean relative photosynthetic photon flux density (rPPFD) for each individual.

### 2.2 Data analyses

After pooling all hermaphrodites (i.e., H, HF, HM, and HFM [hereafter, H‐all]), we compared the sex ratio among the six sexual types using *G* (likelihood ratio) tests between 2010 and 2014. Using temporal changes in the observed sex ratio and the observed transition probability element, the differences in frequencies of sex change among the sexual types (F, M, and H‐all) were evaluated, by calculating a transition probability matrix (3 × 3 matrix). Then, the statistical significance was assessed by conducting 5000 bootstrap runs for the matrices using the “markovchain” package, and steady state was calculated among the sexual types and getting the estimates of the confidence intervals.

To examine the factors affecting the occurrence of sex change, we analyzed the data using generalized linear mixed models (GLMMs) with plot as a random effect (Bolker et al., 2009). The standardized fixed effects were initial individual size (DBH), light environment in both leafless (rPPFD‐winter) and leafy (rPPFD‐summer) seasons, and growth rate. Growth rate per year was measured as the absolute difference between the DBH of the current and previous years. The most appropriate model was selected using Akaike's information criterion (AIC) (Anderson, Burnham, & White, 1998) with backward stepwise selection. We also analyzed the data using Kruskal–Wallis test to examine sexual differences in initial individual size, light environment, and growth rate during 2010–2014 between sex‐changed and constant (no sex‐changed) individuals. Factors influencing the pattern of sex change were also examined to determine the frequency of each focal pattern of sex change using GLMMs, and the best model was selected based on the minimum AIC. Due to insufficient sample size, we analyzed the patterns among females, males, and H‐all for which there were more than 10 changes in sex. The fixed and random effects in the GLMMs were same as in the analysis of the factors affecting the occurrence of sex change. In the analyses examining factors affecting the occurrence and patterns of sex change, the data for the period 2011–2014 were used because no measurements of rPPFD‐winter were available for 2010. All analyses were performed using R ver. 3.1.2 (R development Core Team 2014).

## 3 Results

### 3.1 Patterns of sex change and sex ratio

Of the 309 individuals that were examined in the period 2010–2014, 85 (27.5%) changed their sexes at least once, and 224 (72.5%) never changed sex (Table 1). Several patterns of sex change were observed: 37 individuals (12%) changed sex only once, others changed sex twice (9.7%), three times (5.2%), or in every year over the 5‐year period (0.6%). Sex change was either unidirectional (no reversal to the previous sex) in three patterns, or bidirectional (including reversals to previous sex(es) at least once) in five patterns (Table 1).

**Table 1:** The directions and patterns of sex change in *Eurya japonica* during 2010–2014

Occurence of Sex change	Direction	Pattern	*N* (%)
Constant (no sex change)		A	224 (72.5)
Sex change	Unidirection	A→B	37 (12.0)
		A→B→C	13 (4.2)
		A→B→C→D	2 (0.6)
	Bidirection	A→B→A	17 (5.5)
		A→B→A→B	11 (3.6)
		A→B→A→C	3 (1.0)
		A→B→A→C→D	1 (0.3)
		A→B→C→D→C	1 (0.3)
Total			309

Pattern A→B→A: the sex expression changed from A to B, and then back to A; Pattern A→B→C: the sex expression changed from A to B, and then changed from B to C.

When we reviewed the transition matrix of sex expression between previous and subsequent years for 2010–2014 (in total, 1000 observations), we never observed the following sex changes: F to HM, F to HFM, H to F, H to M, or HFM to F, but we encountered 25 other patterns of sex change among the six sexual types (Table 2). The most frequent patterns of sex change were from H to HF and from HF to F (22 times each during 2010–2014).

**Table 2:** The transition matrices of sex expression between previous and next years in *E*. *japonica* during 2010–2014

Sex of the next year	Sex of the previous year					
	F	M	H	HF	HM	HFM
F	**300**	5	0	22	1	0
M	4	**274**	0	1	6	2
H	1	1	**30**	10	1	1
HF	10	1	22	**168**	2	6
HM	0	10	3	3	**22**	4
HFM	0	3	3	8	3	**10**
Total	315	294	57	212	35	23

The bold letters show that sex did not change.

**Figure 1:**
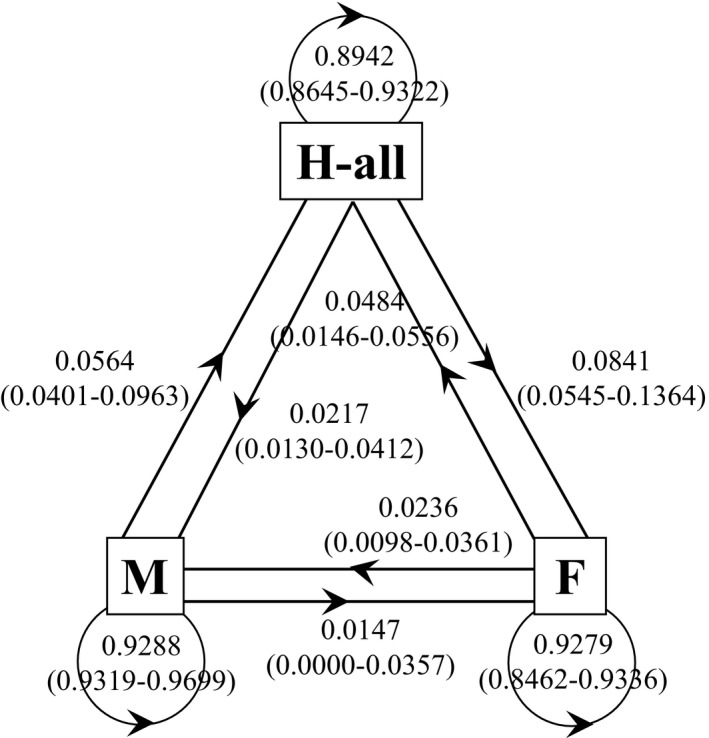
The average and 95% confidence interval (in parentheses) estimated by calculating a transition probability matrix among the sexual types (F, M and H‐all) between previous and next years in *E. japonica*. F, female individuals with only pistillate flowers; M, male individuals bearing only staminate flowers; H‐all, all types of hermaphrodite individuals (H, HF, HM, and HFM; H, hermaphrodite individuals with only perfect flowers; HF, individuals bearing a mixture of perfect and pistillate flowers; HM, individuals bearing perfect and staminate flowers; HFM, individuals bearing a mixture of all three flower types)

According to the transition probability matrix, sex changes from H‐all to F and from M to H‐all tended to occur more frequently than those from F to M and from M to F did, whereas the constant probability (F to F, M to M and H‐all to H‐all) was similar among the three sexual types (Figure 1). Compared to the observed transition matrix, the observed values of F to F (0.9524) and M to M (0.9320) were higher and marginally lower than the estimated values, respectively (Table 2, Figure 1).

**Figure 2:**
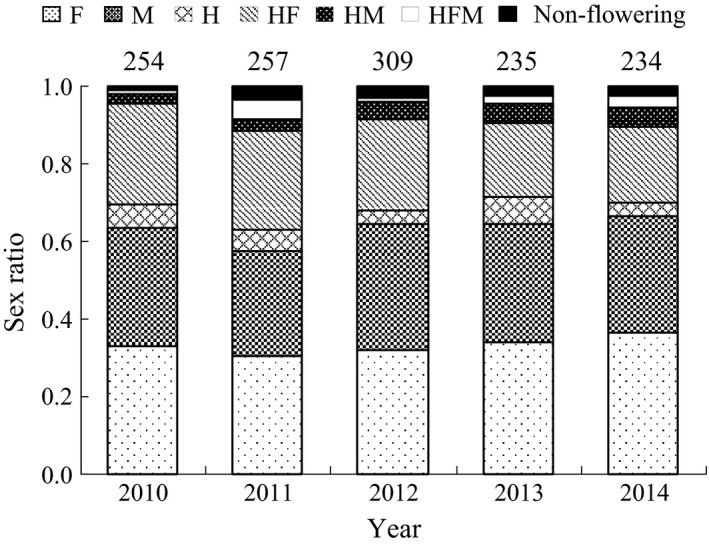
The ratio of six sexual types and non‐flowering in each year from 2010 to 2014 in *E*. *japonica*. The number of individuals is indicated above each bar. For the abbreviation of sexual types, see Figure 1

The average proportions of the six sexual types in the period 2010–2014 were as follows: F, 33.2%; M, 30.1%; H, 5.1%; HF, 22.6%; HM, 4.0%; and HFM, 2.4%. The sex ratio in *E*. *japonica* did not significantly fluctuate from year to year or over the 5 years (Figure 2). Across this time there was no significant difference in the sex ratio between 2010 and 2014 (*G *= 9.45, *p *= .092; Figure 2). When the sex ratio was examined between 2010 and 2014 using only data for plots 2 and 3, where sex expression was observed in all 5 years, we found no difference in the sex ratio (*G *= 8.15, *p *= .145).

The mean steady states of females, males, and H‐all were 0.5012 (0.3451–0.6315, 95% confidence interval), 0.2103 (0.1109–0.3080), and 0.2885 (0.2078–0.4184), respectively. Assuming that the expected female:male:H‐all sex ratio is 0.333:0.333:0.333, the calculated steady state of females significantly exceeded the expected value, whereas that of males was significantly lower than 0.333.

### 3.2 Factors that influenced the occurrence and pattern of sex change

**Figure 3:**
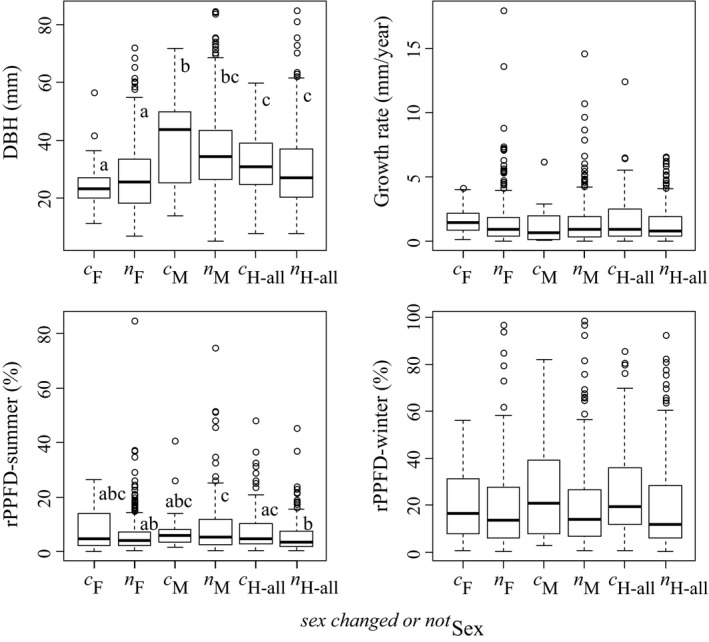
Initial individual size (DBH), the light environments of the leafless (rPPFD‐winter) and leafy (rPPFD‐summer) seasons, and absolute growth rate (mm/year) among initial sex types (F, M, and H‐all) of sex‐changed and non‐changed individuals in each sexual type of *E*. *japonica*. Superscripts of *c* and *n* mean sex‐changed and non‐sex‐changed individuals, respectively. Different letters beside the bars indicate significant differences in the results of multiple comparisons in which family‐wise errors were adjusted using Tukey's method at *p *=* *.05

Although individual size (DBH) (*p *=* *.36), light environments in leafless season (rPPFD‐winter) (*p *=* *.39), and growth rate (*p *=* *.38) did not differ between sex‐changed and non‐changed individuals in each sexual type, sex‐changed H‐all individuals had significantly higher rPPFD‐summer than non‐changed ones (*p < *.02; Figure 3). A significant sexual difference in individual size was also found (*p < *.001) and males were largest and females were smallest (Figure 3).

The explanatory variables included in the model best explaining the occurrence of sex change were the light environments in both seasons (rPPFD‐winter and rPPFD‐summer) and growth rate (Table 3). Sex was more likely to change with higher growth rates and more exposure to light throughout the year. The factors that were frequently selected in the models best explaining the pattern of sex change among females, males, and H‐all were initial individual size, rPPFD‐summer, and growth rate (Table 3). Among individuals that changed sex, those that were less exposed to light in the leafy season and had less diameter growth tended to shift from hermaphrodite to a single sex (i.e., female or male); the smaller individuals changed to female, and the larger individuals changed to male. By contrast, individuals with greater diameter growth were likely to change from a single sex to hermaphrodite. The light environments of both seasons were also selected in the best model for the pattern F to H‐all, and initial individual size and rPPFD‐summer for M to H‐all (Table 3).

**Table 3:** Result of the best model, based on AIC scores, for factors influencing the occurrence and patterns of sex change among females (F), males (M), and all hermaphrodites (H‐all) in *E*. *japonica*. The values in parentheses indicate the frequency of sex change

Model	Size	rPPFD‐winter	rPPFD‐summer	Growth rate
Occurrence of sex change		0.343	0.025	0.164
F to H‐all (15)		−1.278	0.952	0.394
M to H‐all (16)	1.143		−0.911	0.675
H‐all to F (23)	−0.698		−0.106	−0.243
H‐all to M (10)	0.848		−0.988	−1.162

## 4 Discussion

### 4.1 Diverse patterns of sex change but stable sex ratio

We found frequent, repetitive sex changes in subdioecious *E*. *japonica*. Moreover, the sex changes were multidirectional among the six sexual types (25 patterns). This is the first quantitative report on the diverse patterns of sex change in *E*. *japonica*. Tsuji and Sota (2013) described a sex change from hermaphrodite to male, a shift that we never observed. This discrepancy between studies might be explained by the complexity of sex expression in *E*. *japonica*. Sex change from H‐all to male was observed; therefore, if H‐all can be interpreted as synonymous with “hermaphrodite,” our result becomes congruent with the observation by Tsuji and Sota (2013). In our study population, 27.5% of the individuals of subdioecious *E*. *japonica* changed sex at least once, which is a higher frequency of sex change than observed for *Bischofia javanica* (3.7%, Yamashita & Abe, 2002), similar to that in some *Acer rufinerve* populations (11%‐20.7%, Matsui, 1995; Ushimaru & Matsui, 2001), but lower than that in another *A*. *rufinerve* population (54%, Nanami et al., 2004) and in other species, such as *Pinus densiflora* (37%, Kang, 2007) and *Panax trifolium* (57%, Schlessman, 1991). Compared to other subdioecious species, 27.5% is higher in *E*. *japonica* than the frequency of sex change in *Salix myrsinifolia* (5%, Mirski, 2014), and similar to that in *Atriplex canescens* (over 20%, McArthur, 1977; Freeman & McArthur, 1984), but lower than that of *Schiedea globosa* (60%–70%, Sakai & Weller, 1991).

No fluctuation in the sex ratio was detected over the 5 years, although sex changed frequently. The stable sex ratio may result partly from repetitive, bidirectional sex changes in the same individuals (e.g., A→B→A) and partly from complementary changes in sex among individuals (e.g., A→B in one plant and B→A in another). However, comparison between the observed transition matrix and 95% confidence interval of the estimated transition probability matrix suggests that females and males might be gradually increasing and decreasing, respectively, in this *E. japonica* population over the longer period. This hypothesis is also supported by the result of the calculated steady state, in which females significantly exceeded 0.333, whereas males were significantly lower than 0.333. However, it is inconsistent with our previous results that male individuals have an advantage in male fertility over hermaphrodites in hand‐pollinated crosses (Wang, Matsushita, Tomaru, & Nakagawa, 2016). Considering the weakened reproductive success of females versus hermaphrodites under natural conditions in this* E. japonica* population (Wang et al., 2015), pollinator‐mediated interaction and reproductive success through male and female functions may be related to the gradual change in the sex ratio of *E. japonica*.

### 4.2 Sex change in relation to internal condition

Higher frequencies of sex change in *E*. *japonica* were related to a greater growth rate and more abundant illumination throughout the year. However, we found no differences in growth rate and light environments between sex‐changed and non‐changed individuals, except for rPPFD‐summer of H‐all individuals. This suggests that the sex change in *E*. *japonica* results from good internal condition mediated by the light environment. Matsui (1995) and Nanami et al. (2004) also suggested that plant health is coupled with sex change.

The selection of growth rate in each model best explaining the pattern of sex change indicates that the internal physiological condition of an individual *E*. *japonica* is also likely to affect the direction of sex change. Unhealthy conditions induce sex change to the female gender in *Acer* trees (Matsui, 1995; Nanami et al., 2004). In *E*. *japonica*, poor internal condition (i.e., dark light condition in leafy season and reduced growth rate) was linked to sex change from hermaphrodite (H‐all) to single gender status (female or male; Table 3). The difference among sexes in the immediate resource costs of reproduction appears to influence the sex change (Schlessman, 1991). When *E*. *japonica* individuals are in unfavorable condition, such as experiencing inadequate resource availability, they may allocate their limited resources to a single reproductive function. The positive effect of size on the sex change from H‐all to M might be related to sex differences in initial size in which males are larger than females irrespective of the occurrence of sex change. In comparison, female or male individuals in good condition (i.e., high growth rate) may change to hermaphrodites (H‐all) using extra resources to produce additional reproductive organs with a different function. These results in our study population suggest that hermaphrodites (H‐all) are much more likely to be involved with sex change through resource allocation corresponding to internal condition than a single sex. Although the relationship between the pattern of sex change and other factors remains unclear due to their inconsistent effects in the best model, biotic (initial individual size) and abiotic (light environments in both seasons) factors appear to influence the internal condition of *E*. *japonica* individuals. Further experimental approaches are necessary for a more comprehensive understanding.

This study was the first step in exploring sex change and factors that affect the occurrence and pattern of sex change in *E*. *japonica*. Subdioecious *E*. *japonica* was found to have labile sex expression and diverse patterns of sex change. Internal condition was suggested to correlate with the occurrence and pattern of sex change. A constant sex ratio was observed over the 5 years, whereas the estimated transition probability matrix and steady state suggest increasing female and decreasing male individuals over a longer timescale. These findings imply that further studies of *E*. *japonica* will help to elucidate the importance of ecological factors in mediating the sex ratio and sexual system evolution.

## Conflict of Interest

None declared.
