# Supplementary figures and images for: Sex change in the subdioecious shrub Eurya japonica (Pentaphylacaceae)
Source: Ecol Evol. 2017 Mar 10;7(7):2340–5. doi: 10.1002/ece3.2745 (PMC5383483; doi:10.1002/ece3.2745)

## Slide 1
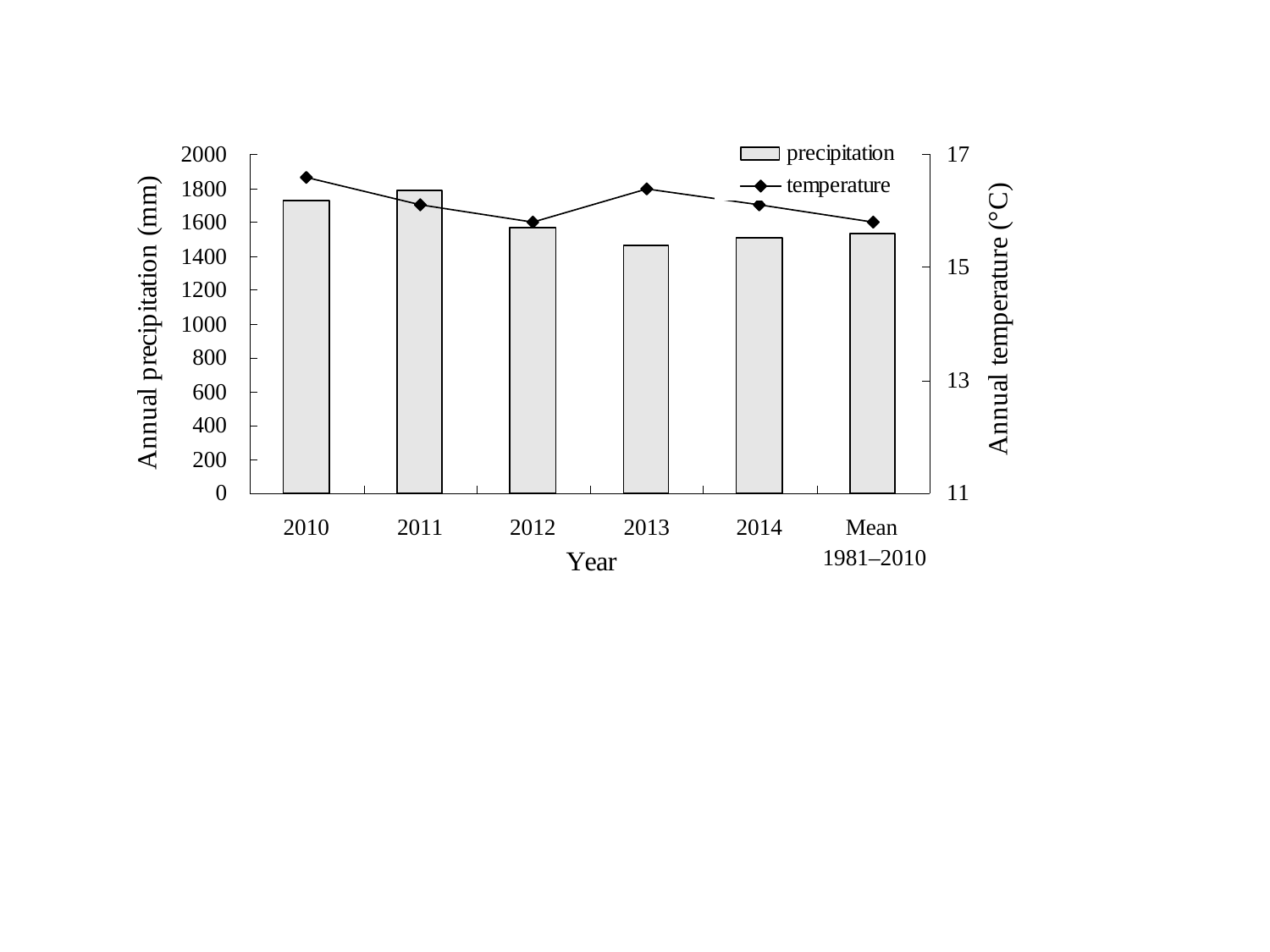

Supplement: Supplementary file 1 [file ECE3-7-2340-s001.ppt]
